# Supplementary material for: Interplay of structural preorganization and conformational sampling in UDP-glucuronic acid 4-epimerase catalysis
Source: Nat Commun. 2024 May 8;15:3897. doi: 10.1038/s41467-024-48281-6 (PMC11519531; doi:10.1038/s41467-024-48281-6)
Supplement: Supplementary file 1 — Supplementary Information [file 41467_2024_48281_MOESM1_ESM.pdf]

Supplementary Information

Interplay of structural preorganization and conformational sampling in UDP-glucuronic acid 4-epimerase catalysis

Christian Rapp[a], Annika Borg[a] and Bernd Nidetzky[a,b,#]

[a] Institute of Biotechnology and Biochemical Engineering, Graz University of Technology, NAWI Graz, 8010 Graz, Austria

[b] Austrian Centre of Industrial Biotechnology (acib), 8010 Graz, Austria

# Corresponding author: bernd.nidetzky@tugraz.at

Supplementary Table 1. Determination of enzyme-bound NADH as isolated (ai) and in the steady-state (ss) at 25 °C with associated UDP-GlcA/UDP-GalA (ligands) using 4 mM UDP-GlcA as the substrate. Conversions to UDP-GalA were within the initial rate period (4.8-6 %).

Variant	NADH (%, ai)	NADH (%, ss)	Enz-UDP-GlcA/ Enz-UDP-GalA	Ligand/enzyme (µM/µM)
WT	3.05 ± 0.10	1.50 ± 0.10	1.70 ± 0.03	0.85 ± 0.02
S127A	4.35 ± 1.36	3.71 ± 0.23	1.64 ± 0.01	0.86 ± 0.03
P85G	5.57 ± 2.10	4.76 ± 3.29	1.71 ± 0.02	0.84 ± 0.16
R88A	1.43 ± 0.70	1.88 ± 0.74	1.73 ± 0.01	0.88 ± 0.06
Y149F	3.44 ± 0.31	≤4	-	-

Supplementary Table 2. DNA primer sequences for *Bc*UGAepi_P85G.

Name	Mutation	DNA primer (5' – 3')
P85G-fwd	P85G	CATCTGGCGGCGATCGGTGGCGTTCGTACCAG
P85G -rev	P85G	CTGGTACGAACGCCACCGATCGCCGCCAGATG

Supplementary Table 3. Experimental and predicted thermodynamic parameters for the temperature dependence of the standard folding free energy ΔG_r for *Bc*UGAepi variants under physiological condition. The crystal structure of *Bc*UGAepi_WT in complex with UDP and NAD (chain A; PDB: 6ZL6) was set as input parameter and variants thereof generated via *in silico* mutation using PyMOL. T_m: melting temperature; ΔH_m: standard folding enthalpy; ΔC_p: standard folding heat capacity; ΔG_r: standard folding free energy at 298.15 K. Predictions were performed using SCooP[1] (http://babylone.ulb.ac.be/SCooP). See Supplementary Fig 7 for melting curves.

*Bc*UGAepi variants	ΔH_m (kcal mol^{-1})	ΔG_r (kcal mol^{-1})	ΔC_p (kcal mol^{-1} K^{-1})	T_m (°C)
WT (exp.)	-	-	-	57 ± 2
WT (predict.)	-116.80	-5.40	-3.50	62.4
Y149F (predict.)	-116.20	-5.60	-3.23	67.1
R88A (predict.)	-116.60	-5.60	-3.23	67.3
S127A (predict.)	-115.90	-5.60	-3.23	67.0
P85G (predict.)	-116.90	-5.60	-3.27	66.6

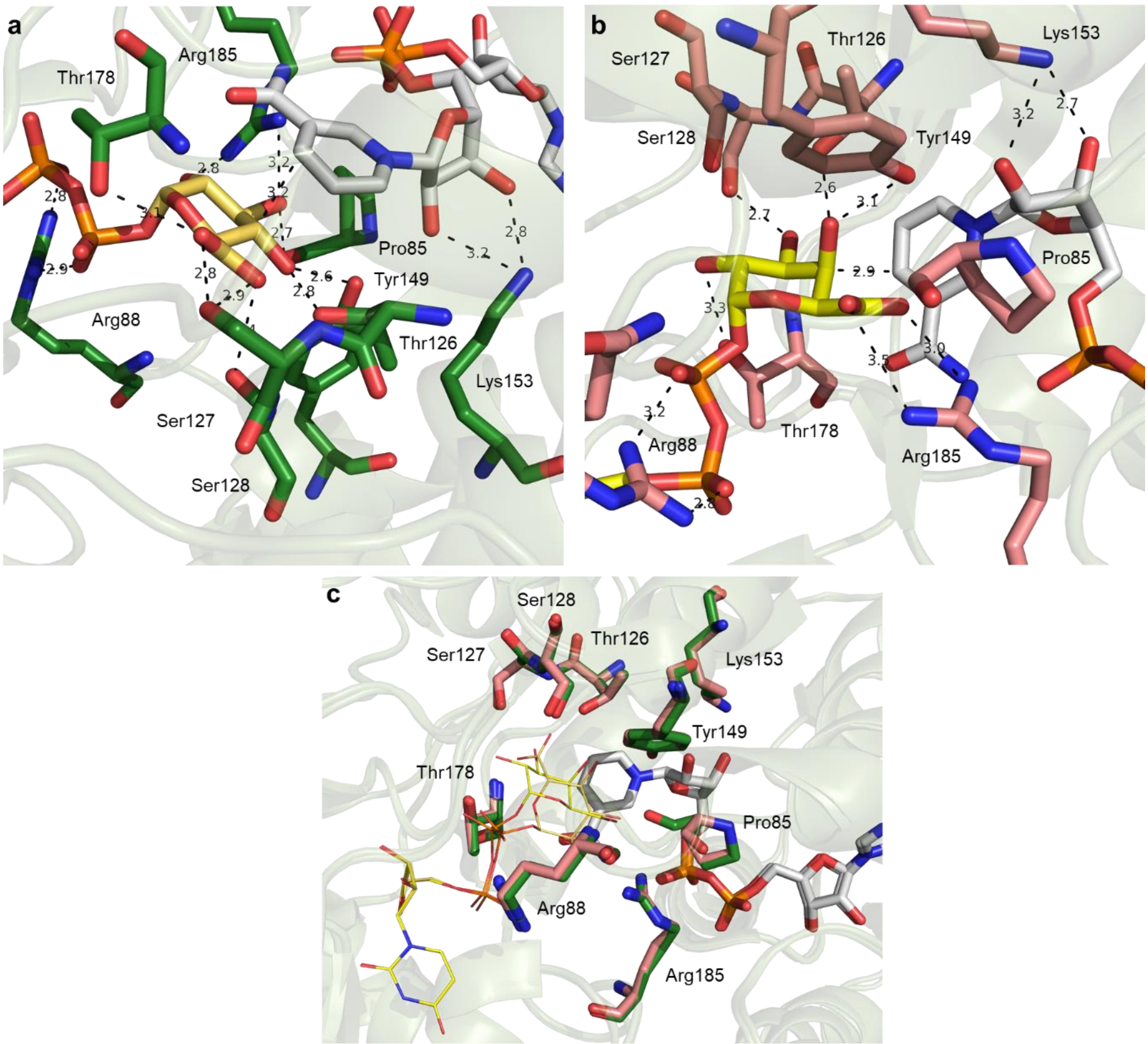

Supplementary Figure 1. Structural comparison of ground-state configurations of *Bc*UGAepi in complex with UDP-GlcA or UDP-GalA. (a) Central interactions in the *gluco*-configuration are shown, with Tyr149, Thr126 and Lys153 forming the catalytic triad. (b) Arg185 clamps and stabilizes the carboxylate in the *galacto*-configuration. (c) Structural overlay of both configurations with flipped Pro85 representing the only change in the active site. Color code: *Gluco*-configuration: residue color: dark green; UDP-GlcA: dark yellow. *Galacto*-configuration: residue color: salmon; UDP-GlcA: bright yellow. NAD^+ coenzyme is shown in grey. See PDB: 6ZLD, 6ZLL and reference[2].

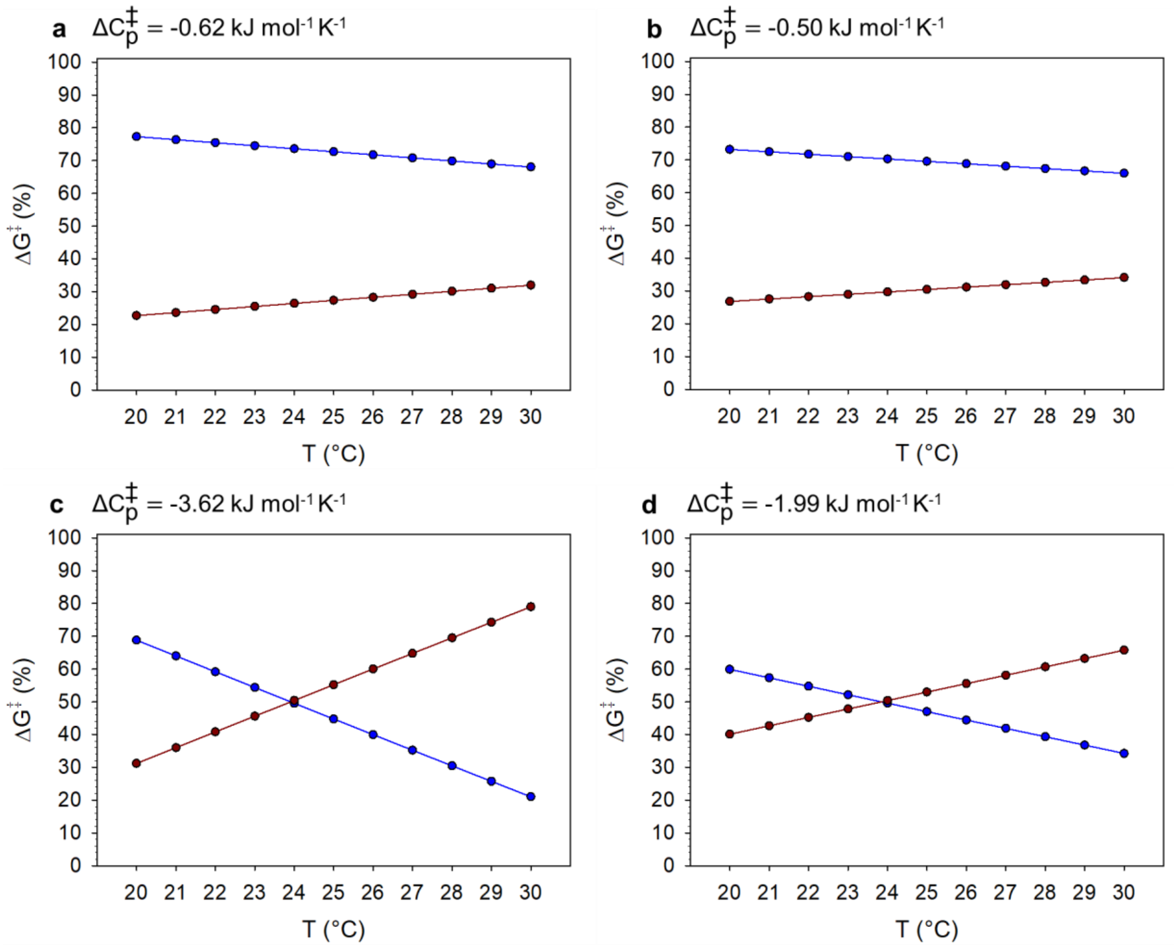

Supplementary Figure 2. Entropy-enthalpy partitioning across a temperature range of 20-30°C in the wild-type enzyme and S127A variant. Effects of temperature on $\Delta H^{\ddagger}$ (%; blue lines) and $\Delta S^{\ddagger}$ (%; red lines) for wild-type reactions with (a) protiated and (b) deuterated substrate are faintly pronounced (< 9.3%). By contrast, the S127A variant displays strong temperature dependence for (c) light and (d) heavy isotope. Corresponding activation heat capacities $\Delta C_p^{\ddagger}$ are displayed above the panels. Total Gibbs free energy barriers $\Delta G^{\ddagger}$ (%) refer to individual calculations from contributions of $\Delta H^{\ddagger}$ and $\Delta S^{\ddagger}$ at the respective temperature.

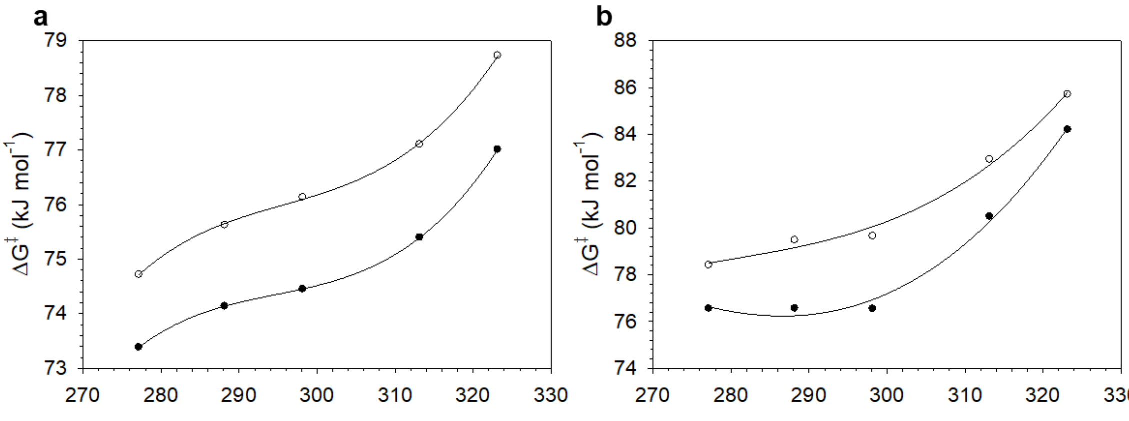

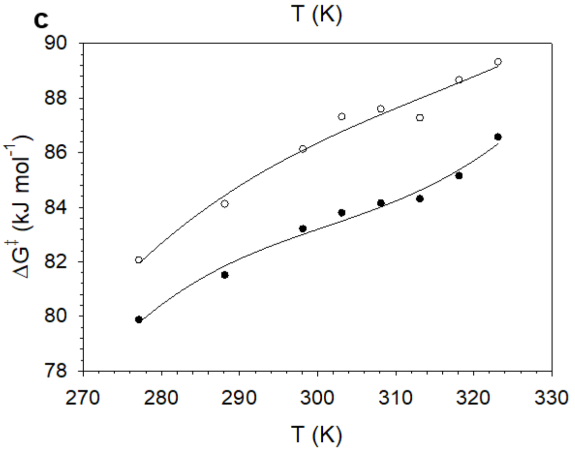

d

Parameter	Wild-type	S127A	P85G
ΔS‡ (J mol-1K-1)	-31.91 (-42.19)[a]	-125.60 (-115.84)[a]	-99.79 (-144.24)[a]
ΔH‡ (kJ mol-1)	64.94 (63.52)[a]	39.49 (45.52)[a]	53.25 (43.09)[a]
$\Delta C_p^{\ddagger}$ (kJ mol-1 K-1)	43.32 (42.47)[a]	16.82 (29.94)[a]	45.84 (19.62)[a]
m (J mol-1 K-2)	-147.01 (-143.66)[a]	-68.37 (-106.82)[a]	-152.28 (-62.33)[a]

[a] Data for the [2H] substrate.

Supplementary Figure 3. Fits of temperature profiles for representative *Bc*UGAepi variants using Supplementary Equation 2. The linear approximation of the two-state MMRT[3] model was applied for fitting data of (a) wild-type, (b) S127A and (c) P85G. Obtained parameters are listed in (d). Closed and open symbols correspond to [^{1}H] and [^{2}H] isotopes, respectively. T_0 was set to 298.15 K.

Discussion (Supplementary Figure 3):

Transition state theory (Supplementary Equation 1) was used to calculate individual values for $\Delta G^{\ddagger}$ from the measured k_{cat}.

$$k_{cat} = \frac{\gamma k_B T}{h} e^{-\Delta G^{\ddagger} / RT} \quad (1)$$

R is gas constant (kJ mol^{-1} K^{-1}), T is temperature (K), γ is transmission coefficient (= 1), $\Delta G^{\ddagger}$ is Gibbs free energy of activation (kJ mol^{-1}), k_B is Boltzmann constant (1.381 × 10^{-23} J K^{-1}) and h is Planck constant (6.626 × 10^{-34} Js).

The limited amount of data points rendered fitting to the five-parameter MMRT-2S model unreasonable.[3] Hence, MMRT-1L involving linear temperature dependence of $\Delta C_p^{\ddagger}$ was used (Supplementary Equation 2).

$$\Delta G^{\ddagger}(T) = \Delta H^{\ddagger}_{T0} - T\Delta S^{\ddagger}_{T0} + \Delta C_p^{\ddagger}\ (T - T_0 - T\ln(T/T_0)) - \frac{m}{2}(T - T_0)^2 \qquad (2)$$

$\Delta G^{\ddagger}$ is Gibbs free energy of activation (kJ mol^{-1}), $\Delta H^{\ddagger}$ is enthalpy of activation (kJ mol^{-1}), $\Delta S^{\ddagger}$ is entropy of activation (kJ mol^{-1} K^{-1}), T is temperature (K) with T_0 being an arbitrary reference temperature (K), $\Delta C_p^{\ddagger}$ is activation heat capacity (kJ mol^{-1} K^{-1}), and m is the slope of $\Delta C_p^{\ddagger}$ representing the size of $\Delta\Delta C_p^{\ddagger}$ in a two-state transition across the temperature range.

Though values obtained for $\Delta S^{\ddagger}$ and $\Delta H^{\ddagger}$ appear plausible physically, m requires interpretation within the context of the model and suggests steep transition for both, wild-type and P85G. Heat capacity is strongly positive for all variants shown and thus conflicts with expectations on magnitude and sign ($\Delta C_p^{\ddagger} \leq 0$).

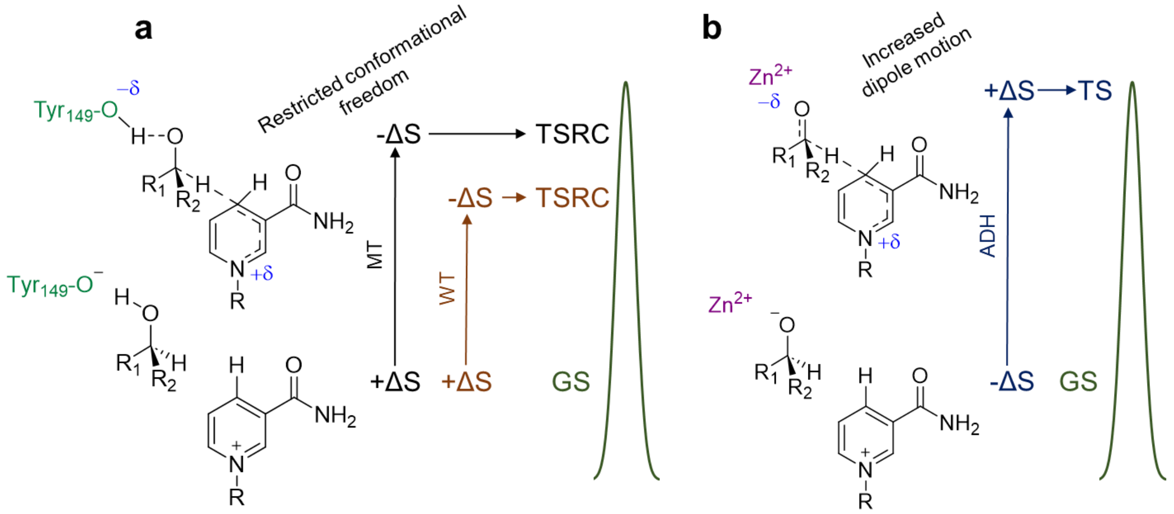

Supplementary Figure 4. Comparative entropic contribution during transition from GS to TSRC/TS in *Bc*UGAepi and ADH. (a) Substrate alcohol deprotonation in *Bc*UGAepi is facilitated by Tyr149 acting as the catalytic base. Note: A pK_a of 5.90 (±0.05) was calculated for Tyr149, which is therefore expected to be ionized at the GS (ground state) due to reactions performed at pH 7.6.[4] Increased entropic penalty for mutants (MT) compared to the wild-type (WT) enzyme is attributed to conformational sampling from a flexible GS to the rigid TSRC (transition state-like reactive conformation) for barrier crossing. (b) The deprotonation step in ADH (alcohol dehydrogenase) is assisted by Zn^{2+} providing electrostatic stabilization in proceeding from a polar GS to the less polar TS (transition state). Inversion of negative to positive ΔS contribution was assumed to arise from increasing dipole motions. See reference[5] for comparison.

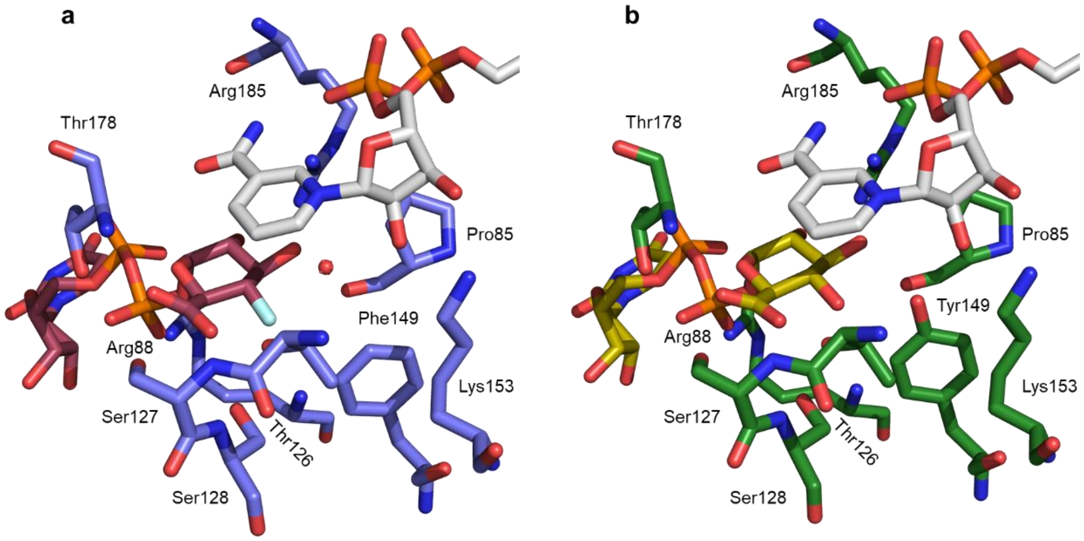

Supplementary Figure 5. Ground state preorganization in the Y149F crystal structure and wild-type enzyme. (a) An ordered water molecule (red sphere) in the Y149F active site enables rescue of the catalytic function. (b) *Bc*UGAepi wild-type preorganization highly resembles the Y149F active site configuration. Color code: residue colors: blue and dark green; UDP-4-deoxy-4-fluoro GlcA and UDP-GlcA are shown in raspberry red and dark yellow, respectively. NAD^+ coenzyme is shown in grey. See PDB: 6ZLJ, 6ZLD and reference[2].

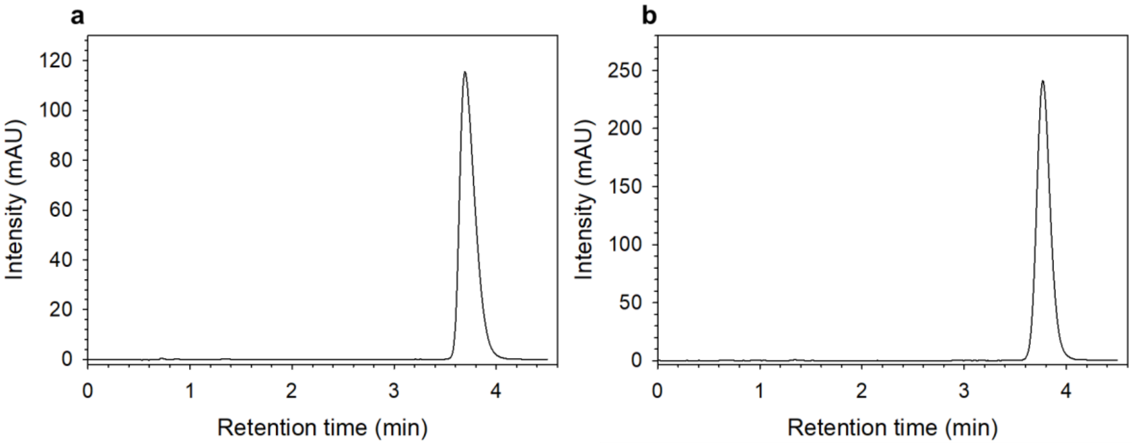

Supplementary Figure 6. HPLC chromatograms of purified UDP-GlcA and UDP-[4-^{2}H]-GlcA. (a) UDP-GlcA and (b) UDP-[4-^{2}H]-GlcA. Products were obtained in ≥ 99.5 % purity (ʎ = 262 nm). mAU = milli-absorbance units.

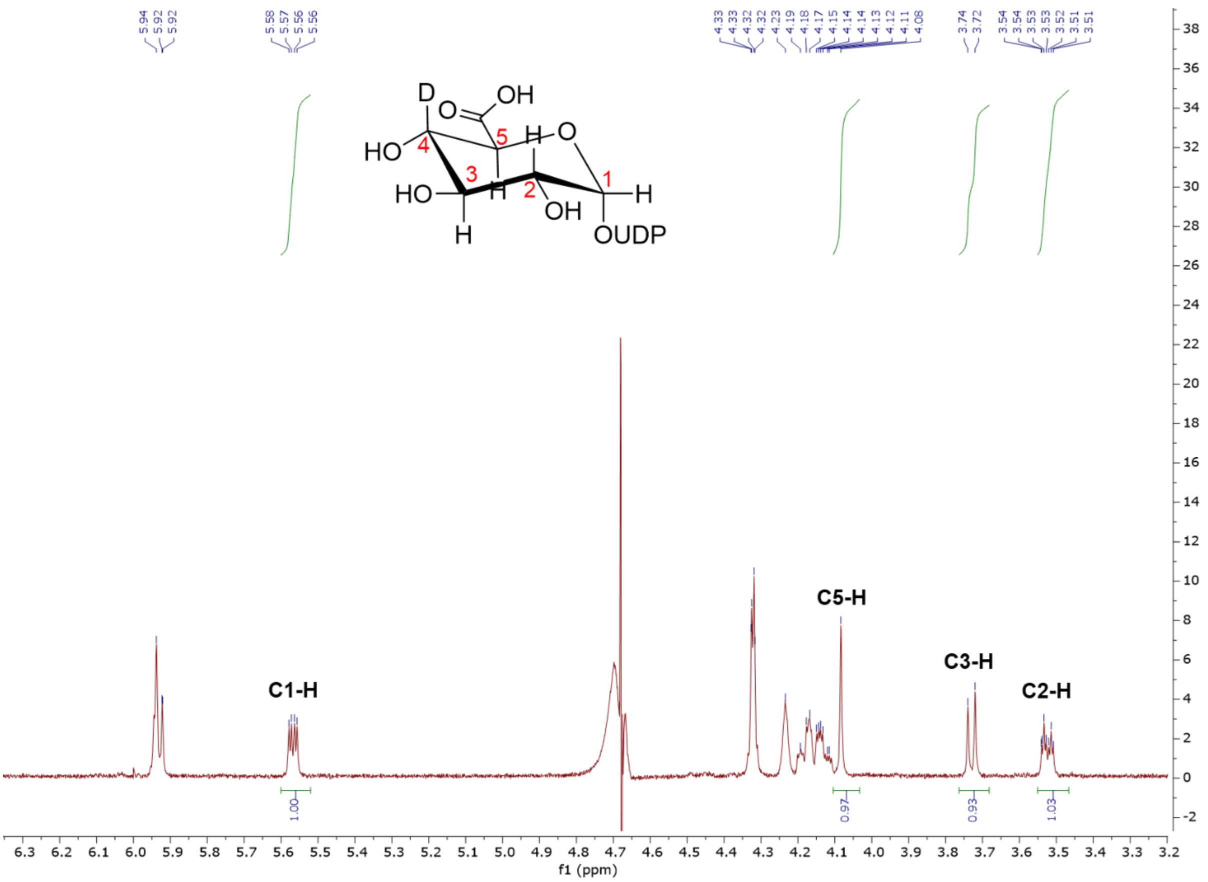

Supplementary Figure 7. ^{1}H NMR spectrum (500 MHz, D_2O) of purified UDP-[4-^{2}H]-GlcA. δ 5.57 ppm (C1-H, dd, 1H), 4.08 ppm (C5-H, s, 1H), 3.73 ppm (C3-H, d, 1H), 3.53 ppm (C2-H, dd, 1H).

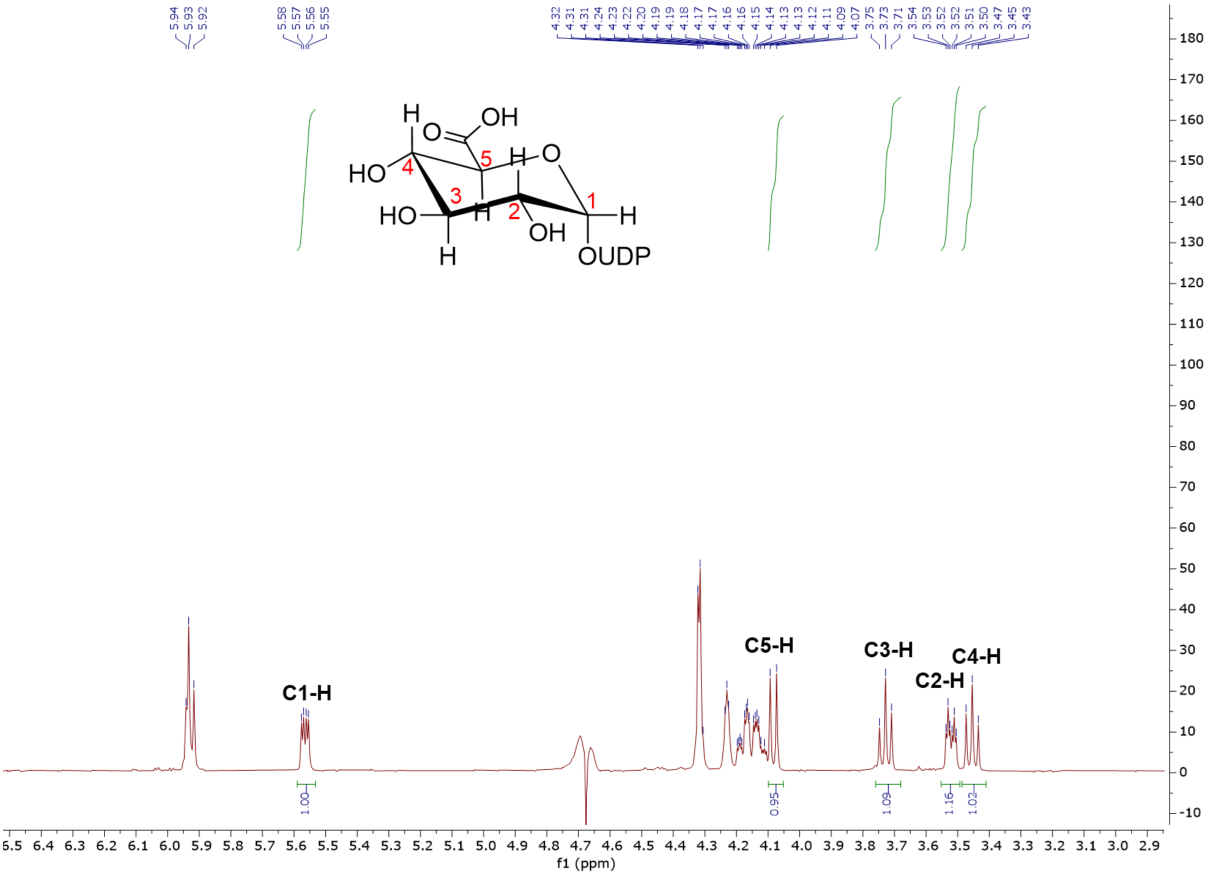

Supplementary Figure 8. ^{1}H NMR spectrum (500 MHz, D_2O) of purified UDP-GlcA. δ 5.57 ppm (C1-H, dd, 1H), 4.08 ppm (C5-H, d, 1H), 3.73 ppm (C3-H, t, 1H), 3.52 ppm (C2-H, dd, 1H), 3.46 ppm (C4-H, t, 1H).

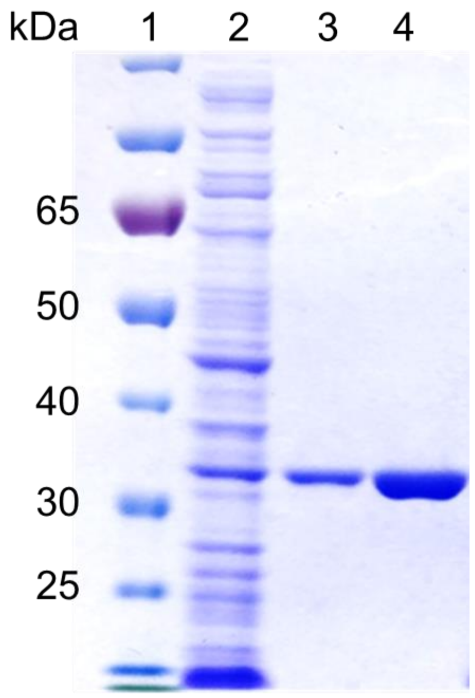

Supplementary Figure 9. SDS polyacrylamide gel showing the purified BcUGAepi_P85G. Lane 1: molecular mass ladder; lanes 2 & 3: Strep-tag affinity chromatography flowthrough and washing fractions; lane 4: concentrated BcUGAepi_P85G (MW = 36977 g mol^{-1}).

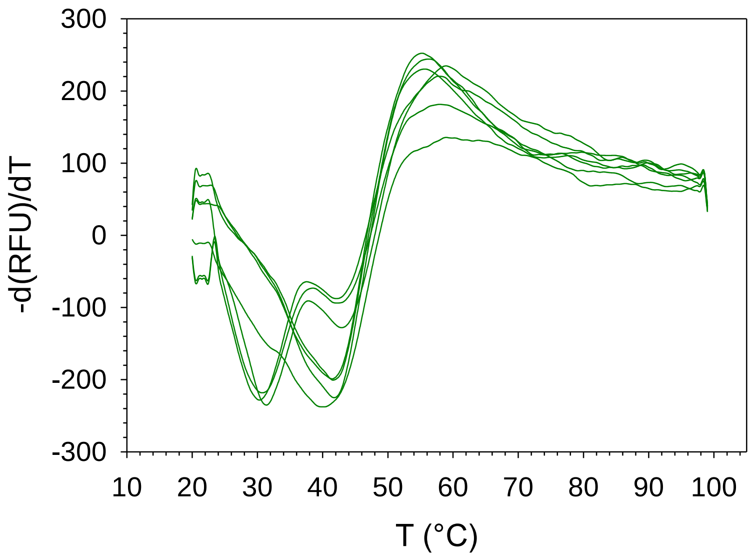

Supplementary Figure 10. Collective melting curves recorded for *Bc*UGAepi. Data was processed by the CFX Maestro software resulting in similar melting temperatures T_m (55–59 °C) for all buffer systems used. See Supplementary Table 3 for T_m predictions and Materials & Methods for experimental description.

References

1. Pucci, F., Kwasigroch, J. M. & Rooman, M. SCooP: An Accurate and Fast Predictor of Protein Stability Curves as a Function of Temperature. *Bioinformatics* **33**, 3415–3422 (2017).
2. Iacovino, L. G. *et al.* Crystallographic Snapshots of UDP-Glucuronic Acid 4-Epimerase Ligand Binding, Rotation, and Reduction. *J. Biol. Chem.* **295**, 12461–12473 (2020).
3. Walker, E. J. *et al.* Cooperative Conformational Transitions Underpin the Activation Heat Capacity in the Temperature Dependence of Enzyme Catalysis. *ACS Catal.* **14**, 4379–4394 (2024)
4. Borg, A., Dennig, A., Weber, H. & Nidetzky, B. Mechanistic Characterization of UDP-Glucuronic Acid 4-Epimerase. *FEBS J.* **288**, 1163–1178 (2021).

5. Roy, S., Schopf, P. & Warshel, A. Origin of the Non-Arrhenius Behavior of the Rates of Enzymatic Reactions. *J. Phys. Chem. B* **121**, 6520–6526 (2017).
