# Supplementary material for: Interplay of structural preorganization and conformational sampling in UDP-glucuronic acid 4-epimerase catalysis
Source: Nat Commun. 2024 May 8;15:3897. doi: 10.1038/s41467-024-48281-6 (PMC11519531; doi:10.1038/s41467-024-48281-6)
Supplement: Supplementary file 3 — Reporting Summary [file 41467_2024_48281_MOESM3_ESM.pdf]

nature portfolio

Corresponding author(s): Bernd Nidetzky

Last updated by author(s): Apr 19, 2024

Reporting Summary

Nature Portfolio wishes to improve the reproducibility of the work that we publish. This form provides structure for consistency and transparency in reporting. For further information on Nature Portfolio policies, see our Editorial Policies and the Editorial Policy Checklist.

Statistics

For all statistical analyses, confirm that the following items are present in the figure legend, table legend, main text, or Methods section.

n/a	Confirmed	
☐	☒	The exact sample size (n) for each experimental group/condition, given as a discrete number and unit of measurement
☐	☒	A statement on whether measurements were taken from distinct samples or whether the same sample was measured repeatedly
☒	☐	The statistical test(s) used AND whether they are one- or two-sided<br>*Only common tests should be described solely by name; describe more complex techniques in the Methods section.*
☒	☐	A description of all covariates tested
☒	☐	A description of any assumptions or corrections, such as tests of normality and adjustment for multiple comparisons
☐	☒	A full description of the statistical parameters including central tendency (e.g. means) or other basic estimates (e.g. regression coefficient) AND variation (e.g. standard deviation) or associated estimates of uncertainty (e.g. confidence intervals)
☒	☐	For null hypothesis testing, the test statistic (e.g. F, t, r) with confidence intervals, effect sizes, degrees of freedom and P value noted<br>*Give P values as exact values whenever suitable.*
☒	☐	For Bayesian analysis, information on the choice of priors and Markov chain Monte Carlo settings
☒	☐	For hierarchical and complex designs, identification of the appropriate level for tests and full reporting of outcomes
☒	☐	Estimates of effect sizes (e.g. Cohen's d, Pearson's r), indicating how they were calculated

Our web collection on statistics for biologists contains articles on many of the points above.

Software and code

Policy information about availability of computer code

Data collection	Molecular mass and extinction coefficients for proteins were calculated using the ProtParam tool (https://web.expasy.org/protparam/). LabSolutions v5.6 from Shimadzu was used for HPLC operation. CFX Maestro software 1.0 (Bio-Rad) was used for measuring melting curves. Prediction of protein melting temperatures was performed using SCooP (http://babylone.ulb.ac.be/SCooP). Proton NMR spectra were recorded on a Varian INOVA 500-MHz NMR spectrometer (Agilent Technologies, Santa Clara, California, USA) using the VNMRJ 2.2D software.
Data analysis	LabSolutions v5.6 from Shimadzu was used for HPLC data analysis. PyMOL Molecular Graphics System v2.5.4 (Open-Source, Schrödinger, LLC) was used for structural depictions. SigmaPlot v15.0 was used for data fitting. CFX Maestro software 1.0 (Bio-Rad) was used for analyzing melting curves. NMR spectra were analyzed using MestReNova 16.0 (Mestrelab Research, S.L., Santiago de Compostela, Spain).

For manuscripts utilizing custom algorithms or software that are central to the research but not yet described in published literature, software must be made available to editors and reviewers. We strongly encourage code deposition in a community repository (e.g. GitHub). See the Nature Portfolio guidelines for submitting code & software for further information.

Data

Policy information about availability of data

All manuscripts must include a data availability statement. This statement should provide the following information, where applicable:

- Accession codes, unique identifiers, or web links for publicly available datasets
- A description of any restrictions on data availability
- For clinical datasets or third party data, please ensure that the statement adheres to our policy

All data are available from the corresponding author (B.N.) upon request. The authors declare that all data supporting the findings of this study are available within the article and its Supplementary Information. Additionally, data for Figures 2, 3 and Supplementary Figure 9 are provided in the Source Data file. All crystal structures used in this study are available in the RCSB Protein Data Bank under following PDB identifiers: 6ZLL [https://doi.org/10.2210/pdb6ZLL/pdb], 6ZLD [https://doi.org/10.2210/pdb6ZLD/pdb], 6ZLJ [https://doi.org/10.2210/pdb6ZLJ/pdb].

Research involving human participants, their data, or biological material

Policy information about studies with human participants or human data. See also policy information about sex, gender (identity/presentation), and sexual orientation and race, ethnicity and racism.

Reporting on sex and gender	n/a
Reporting on race, ethnicity, or other socially relevant groupings	n/a
Population characteristics	n/a
Recruitment	n/a
Ethics oversight	n/a

Note that full information on the approval of the study protocol must also be provided in the manuscript.

Field-specific reporting

Please select the one below that is the best fit for your research. If you are not sure, read the appropriate sections before making your selection.

[x] Life sciences [ ] Behavioural & social sciences [ ] Ecological, evolutionary & environmental sciences

For a reference copy of the document with all sections, see nature.com/documents/nr-reporting-summary-flat.pdf

Life sciences study design

All studies must disclose on these points even when the disclosure is negative.

Sample size	Sample sizes are reported in the manuscript and in the legend of the respective figure. No statistical method was used for sample size determination. The common study design in most published articles involves three replicates with sufficient reproducibility to be considered as representative.
Data exclusions	No data were excluded.
Replication	Three independent and successful replicates were performed for all experiments.
Randomization	Randomization was irrelevant to this study, since absence of a population (participants, medical or environmental samples) exlcudes the generation of a selection bias.
Blinding	Blinding was not relevant to this study, since there is no subjective bias in data analysis or experimental setup.

Reporting for specific materials, systems and methods

We require information from authors about some types of materials, experimental systems and methods used in many studies. Here, indicate whether each material, system or method listed is relevant to your study. If you are not sure if a list item applies to your research, read the appropriate section before selecting a response.

Materials & experimental systems

n/a	Involved in the study
☒	☐ Antibodies
☒	☐ Eukaryotic cell lines
☒	☐ Palaeontology and archaeology
☒	☐ Animals and other organisms
☒	☐ Clinical data
☒	☐ Dual use research of concern
☒	☐ Plants

Methods

n/a	Involved in the study
☒	☐ ChIP-seq
☒	☐ Flow cytometry
☒	☐ MRI-based neuroimaging

Plants

Seed stocks	n/a
Novel plant genotypes	n/a
Authentication	n/a
